# Impact of PGL-I Seropositivity on the Protective Effect of BCG Vaccination among Leprosy Contacts: A Cohort Study

**DOI:** 10.1371/journal.pntd.0001711

**Published:** 2012-06-19

**Authors:** Nádia C. Düppre, Luiz Antonio B. Camacho, Anna M. Sales, Ximena Illarramendi, José Augusto C. Nery, Elizabeth P. Sampaio, Euzenir N. Sarno, Samira Bührer-Sékula

**Affiliations:** 1 Leprosy Unit, Oswaldo Cruz Foundation, Fiocruz, Rio de Janeiro, Brazil; 2 Department of Epidemiology, Sergio Arouca National Public Health School, Fiocruz, Rio de Janeiro, Brazil; 3 Tropical Pathology and Public Health Institute, Federal University of Goiás, Goiânia, Brazil; University of California San Diego School of Medicine, United States of America

## Abstract

**Background:**

Contacts of leprosy patients are at increased risk of developing leprosy and need to be targeted for early diagnosis. Seropositivity to the phenolic glycolipid I (PGL-I) antigen of *Mycobacterium leprae* has been used to identify contacts who have an increased risk of developing leprosy. In the present study, we studied the effect of seropositivity in patient contacts, on the risk of developing leprosy, stratified by Bacille Calmette Guerin (BCG) vaccination after index case diagnosis.

**Methodology/Principal Findings:**

Leprosy contacts were examined as part of the surveillance programme of the Oswaldo Cruz Institute Leprosy Outpatient Clinic in Rio de Janeiro. Demographic, social, epidemiological and clinical data were collected. The presence of IgM antibodies to PGL-I in sera and BCG vaccination status at the time of index case diagnosis were evaluated in 2,135 contacts. During follow-up, 60 (2.8%; 60/2,135) leprosy cases were diagnosed: 41 among the 1,793 PGL-I-negative contacts and 19 among the 342 PGL-I-positive contacts. Among PGL-I-positive contacts, BCG vaccination after index case diagnosis increased the adjusted rate of developing clinical manifestations of leprosy (Adjusted Rate Ratio (aRR) = 4.1; 95% CI: 1.8–8.2) compared with the PGL-I-positive unvaccinated contacts (aRR = 3.2; 95% CI: 1.2–8.1). The incidence density was highest during the first year of follow-up for the PGL-I-positive vaccinated contacts. However, all of those contacts developed PB leprosy, whereas most MB cases (4/6) occurred in PGL-I-positive unvaccinated contacts.

**Conclusion:**

Contact examination combined with PGL-I testing and BCG vaccination remain important strategies for leprosy control. The finding that rates of leprosy cases were highest among seropositive contacts justifies targeting this specific group for close monitoring. Furthermore, it is recommended that PGL-I-positive contacts and contacts with a high familial bacteriological index, regardless of serological response, should be monitored. This group could be considered as a target for chemoprophylaxis.

## Author Summary

Although leprosy has become a neglected disease, it is an important cause of disability, and 250,000 new cases are still diagnosed worldwide every year. The current study was carried out in Brazil, where almost 40,000 new cases of leprosy are diagnosed every year. The study targeted contacts of leprosy patients, who are at the highest risk of contracting the disease. We studied 2,135 contacts who were diagnosed at the Leprosy Outpatient Clinic at the Oswaldo Cruz Foundation in Rio de Janeiro, RJ, Brazil, between 1987 and 2007. The presence of antibodies against a specific *Mycobacterium leprae* antigen (PGL-I) at the first examination and BCG vaccination status were evaluated. PGL-I-positive contacts had a higher risk of developing leprosy than PGL-I-negative contacts. Among the former, vaccinated contacts were at higher risk than unvaccinated contacts. Our results indicate that contact examination combined with PGL-I testing and BCG vaccination appears to justify the targeting of PGL-I-positive individuals for enhanced surveillance. Furthermore, it is highly recommended that PGL-I-positive contacts and contacts with a high familial bacterial index (i.e., the sum of results from index and co-prevalent cases), regardless of serological response, should be monitored. This group could be considered as a target for chemoprophylaxis.

## Introduction

It was widely expected that the treatment of all newly diagnosed leprosy cases with multidrug therapy (MDT) would not only cure the disease but also prevent the further spread of *Mycobacterium leprae (M. leprae)*. In fact, from 2004 to 2010, the number of newly diagnosed cases worldwide fell by 44%. Nonetheless, incidence rates above 1 case in 10,000 remain in a few countries, namely Brazil, Nepal, Liberia, and a few islands in the Western Pacific. Brazil reported the most cases in the Americas and the second most worldwide in 2010 [1]. Of the 34,894 new leprosy patients diagnosed in Brazil in 2010, 5.36% were children under 15, an indication that the transmission of *M. leprae* is ongoing [2]. Notably, 7.2% of the newly detected leprosy cases were of disability grade 2 [3], demonstrating the heretofore limited effectiveness of case detection and the magnitude of the hidden prevalence of leprosy [4].

Maintenance of poverty and of the intensity of exposure may have contributed to the low effectiveness of leprosy control programs. Even if the effectiveness of case detection and MDT treatment could be improved, additional interventions would be needed, with a special focus on groups at particularly high risk of developing clinical leprosy [5]. It has long been known that contacts of leprosy patients have an increased risk of developing leprosy compared with the general population [6].

The detection of antibodies to the phenolic glycolipid I (PGL-I) antigen of *M. leprae* has been used to understand the epidemiology of subclinical infection, as opposed to active disease. However, this technique has not been proven for the early diagnosis of clinical cases and for predicting who (either among contacts of known cases or among the general population) will develop clinical leprosy in the future [7]. The relationship between PGL-I seroprevalence and the leprosy burden depends on the population studied [8], [9]. Seropositivity has been reported to be higher in contacts of leprosy patients than among the general population and has been associated with the development of leprosy [10], [11]. Although PGL-I (−) based serological tests cannot be used as screening tools in the general population, they have been used to identify contacts of leprosy patients who have a higher risk of developing leprosy [12]. After 7 years of follow-up of a cohort of 559 household contacts of multibacillary (MB) patients with a bacteriological index (BI) greater than or equal to 2, Douglas *et al.* [13] reported that seropositive (PGL-I (+)) contacts in the Philippines had a 7-fold higher risk of developing leprosy compared with seronegative (PGL-I (−)) contacts. The Yalisombo Study Group [14] reported a slightly higher proportion of cases among PGL-I (+) (1/189; 0.53%) compared with PGL-I (−) contacts (10/3018; 0.33%) in a survey of 4 hyperendemic villages in Zaire. Other studies have not reported an increased risk of developing leprosy among seropositive contacts [15], [16].

The Brazilian Leprosy Control Program has recommended that all household contacts of leprosy patients be examined and receive Bacille Calmette Guerin (BCG) immunization as an additional preventive measure against leprosy [17]. According to Bagshawe *et al.* [18], the accelerated manifestations of benign tuberculoid leprosy after BCG vaccination reflect BCG vaccination acceleration of the natural history of *M. leprae* infection in individuals who were infected prior to or immediately after vaccination. In line with this result, Duppre *et al.* [19] found that vaccinated contacts contracted leprosy mainly from MB index cases (ICs), suggesting the presence of subclinical infection which becomes overt due to vaccination induced immune response activation.

Because previous studies have failed to reach a consensus regarding the effect of seropositivity on the risk of developing leprosy among contacts and the degree of protection conferred by prior BCG vaccination (BCG scar), further investigation seemed necessary. Thus, the effects of simultaneous BCG vaccination and other possible covariates on the diagnosis of overt leprosy were studied in a group of contacts participating in a surveillance program. The effect of PGL-I seropositivity in contacts, adjusted by covariates measured at the first examination, on the risk of developing leprosy was assessed *per se* and according to BCG vaccination status after IC diagnosis.

## Materials and Methods

### Study design

This dynamic cohort study was based on the contact surveillance programme of leprosy patients who were diagnosed at the Leprosy Outpatient Clinic of the Oswaldo Cruz Institute, FIOCRUZ, in Rio de Janeiro, RJ, Brazil. Among the 6,060 contacts examined between June 1987 and December 2007, 2,135 (35.2%) were tested for IgM antibodies to PGL-I. During this period, 2.2% (46/2,135) of the contacts were diagnosed at the initial examination (co-prevalent cases), did not receive the BCG vaccine and were excluded from the present study. The subsample of contacts selected for the present study was similar to the contacts not selected in terms of gender (p = 0.61), operational classification of IC (p = 0.87) and presence of BCG vaccination scar (p = 0.98). However, the selected group of contacts was significantly older than those contacts who were not selected (p<0.001). The possible selection bias toward older contacts could be due to the difficulties of blood sampling in children. Most parents refused to allow the collection of blood from their children. Blood sampling in young children became part of the protocol after the introduction of the ML Flow test, which uses only one drop of blood for testing.

The presence of anti-PGL-I antibodies at the first examination was the primary variable of interest. The modifying effect of BCG vaccination after IC diagnosis was highlighted because of its known association with the study outcome.

Household contacts were defined as individuals who lived in the same dwelling (i.e., sharing the same kitchen or social/recreational area). Non-household contacts were defined as those indicated by the IC as having had other types of associations, such as next-door neighbors, blood relatives, friends and colleagues. The duration of association with the IC was not considered during the selection of contacts.

### Contact examination

After confirmation of the leprosy diagnosis, patients were given educational information about the disease, and medical visits were scheduled for their close contacts (within and outside of the household). During the initial visit, contacts were interviewed by a social worker to obtain demographic and social information (e.g., schooling and individual and family income) and the degree of closeness to the IC. All contacts received health education on leprosy and were instructed to report to the Leprosy Outpatient Clinic if any clinical signs of leprosy occurred. In addition, contacts were instructed to visit the Center once a year for a period of 3–5 years. In general, contacts were followed for at least 2 years, and the follow-up period ended in December 2009.

An experienced clinical dermatologist examined all of the contacts to identify any leprosy lesions and the typical BCG vaccine scar. In addition, a neurological exam of peripheral nerves was performed by a qualified physiotherapist or neurologist. If a contact presented signs and symptoms suggestive of leprosy, he or she was assessed by bacteriological, histopathological, and immunological tests. If leprosy was diagnosed, the individual was classified according to the Ridley and Jopling scale [20] and grouped for treatment according to the bacteriological index (BI) results as either multibacillary (MB - positive BI) or paucibacillary (PB - negative BI).

### BCG vaccination

Since 1991, the BCG vaccine has been administered to all healthy contacts, as recommended by the Brazilian Leprosy Control Program [21]; however, 248 (12.8%) of the contacts in the sample group were not vaccinated at their first visit due to pregnancy, acute disease or vaccine shortage. These contacts were rescheduled for vaccination, but 179 (111 of whom had a BCG vaccine scar and 68 of whom had no visible scar) failed to return for vaccination. These noncompliant cases, together with 200 contacts examined before 1991 (104 with a BCG scar and 96 without), were included in the study as part of the unvaccinated group. Thus, among the 2,135 contacts included in this study, 1,756 received simultaneous BCG vaccine at the time of IC diagnosis, and 379 did not.

### PGL-I serology

Before vaccination, blood samples were collected, and the sera were separated into aliquots, followed by storage at −20°C to later determine the presence/absence of anti-PGL-I antibodies (all contacts were eligible).

Two different rapid tests were used for evaluation of the presence of antibodies in blood serum. The ML Dipstick assay [22] was used between 1987 and 2002 to test 1,050 contacts. Beginning in 2003, the ML Flow test was implemented as part of the routine contact examination, and 1,085 contacts were tested in this manner.

The visual readings of both tests were performed as previously described [22]. A reddish-stained antigen band indicated a positive reaction. Both tests presented a high level of agreement in the detection of IgM antibodies to PGL-I using the enzyme-linked immunosorbent assay (ELISA) (97.2% [k = 0.92] and 91% [k = 0.77] for the ML Dipstick and ML Flow tests, respectively) [12].

### Case detection

For the purposes of this study, those contacts who did not return for evaluation were considered free of leprosy. However, due to the low participation of contacts in re-examination during the study period (29%), a complementary strategy was adopted. To ascertain the existence of leprosy contacts who might have moved away or visited another health center, the Brazilian Information System for Notifiable Diseases (SINAN) database was searched for new cases. Reporting cases to the SINAN is compulsory for all municipalities in Brazil and is performed on a weekly basis. The data feed is monitored at the state and national levels according to specific parameters.

SINAN records published in 2010 (i.e., with data on new cases up to 2009) were matched to the database of the present study by the contact's full name, date of birth, and mother's full name. As a result of this search, 3 contacts in the SINAN database were included in the sample group as new leprosy cases.

### Ethics statement

After receiving educational information about leprosy, all adult participants and the guardians or parents of the child participants provided written consent. A medical history for each contact was taken from routine care medical records. Data collection, management, and analysis were performed by the study coordinators, and confidentiality was maintained throughout the research. The present study, including the use of patient records, was approved by the Research Ethics Committee of the National School of Public Health (Document N°. 113/06).

### Statistical analysis

The leprosy incidence rate at the contact follow-up was based on person-years (PYs) between the first examination of a contact and the date of his or her leprosy diagnosis. Contacts who did not return for follow-up or who were not found in the SINAN database were considered to be free of leprosy at the end of the study.

The total familial BI was derived from the sum of all BIs of MB cases in the family at the time of the first examination, which was believed to be a better proxy of disease risk for the contacts who were followed up after the initial examination.

Contacts who did not receive the BCG vaccine after the IC's diagnosis were considered unvaccinated, and those who were vaccinated subsequent to the IC's diagnosis were considered vaccinated. Accordingly, based on their vaccination status and serological response to PGL-I, the contacts were grouped into the following categories: **PVC**, Positive Vaccinated Contacts; **NVC**, Negative Vaccinated Contacts; **PUC**, Positive Unvaccinated Contacts; and **NUC**, Negative Unvaccinated Contacts.

The association between covariates and seropositivity was analyzed using univariate and multivariate logistic regression to generate odds ratios (ORs). Crude and adjusted rate ratios (RRs) were estimated by Poisson regression to verify the association between seropositivity and the development of leprosy, both overall and stratified according to vaccination status. RR estimates were adjusted for age, gender, presence of BCG scar, type of association with the IC, duration of close association with the IC, and sum of the family BIs. The 95% confidence intervals (CIs) were determined for all estimates. Multivariate analyses and CIs were based on robust variance estimators using clusters of contacts. To account for the clustering effect in both types of regressions, the CI was based on robust variance estimators that account for a smaller variance of contacts clustered around the IC. Statistical interaction (RR test of homogeneity) was assessed when judged scientifically meaningful according to the Mantel-Haenszel test.

Statistical analysis was performed with Stata™ version 8.0 (Stata Corp., College Station, TX,USA) and Open Source Epidemiologic Statistics for Public Health version 2.3.1 (http://www.openepi.com/OE2.3/Menu/OpenEpiMenu.htm).

## Results

The present study included 2,135 contacts of 668 ICs (220 PB and 448 MB, with an average of 3.5 contacts per MB patient and 2.6 per PB patient) who were tested for the presence of IgM antibodies to PGL-I from 1987 to 2009. Most of the contacts (1,253; 59%) were female. The mean age was 28.8 (SD: 17.0) years. Most of the contacts (64%) had a low monthly family income (below four minimum salaries defined by law and adjusted periodically according to inflation.

There were no demographic differences between the vaccinated and unvaccinated contacts. In both groups, females predominated (58.3% of vaccinated contacts and 60.4% of unvaccinated contacts). The mean age was significantly greater (t-test = 2.04; p = 0.042) in vaccinated contacts (29.2±17.2 years) compared with unvaccinated contacts (27.2±16.2 years).

Overall, the rate of seropositivity to PGL-I at the first evaluation was 16.0% among contacts. Adjusting for relevant covariates, seropositivity was more frequent among household contacts, females, contacts aged 15–35 years, and contacts with a high family BI. The presence of a BCG scar from prior vaccination, the duration of close association with the IC and the operational classification of the IC did not appear to influence PGL-I positivity (Table 1).

**Table 1:** Crude and adjusted measures of association of seropositivity and selected covariates among leprosy contacts.

	Serology anti PGL-I			Odds ratio (95% C.I.)^3^	
Covariates	n	PGL-I (+) (n%)	p value^1^	Unadjusted	Adjusted^2^
**All contacts Previous BCG scar**	2,135	342 (16.0)	-	-	-
• Without BCG scar	782	108 (13.8)		1	1
• With BCG scar	1,353	234 (17.3)	0.03	1.3 (1.0–1.7)	0.9 (0.6–1.2)
**Gender**					
Male	882	120 (13.6)		1	1
• Female	1,253	222 (17.7)	0.01	1.4 (1.1–1.7)	1.4 (1.1–1.8)
**Age group**					
• 0–14 years	494	73 (14.8)		1.2 (0.9–1.8)	1.1 (0.7–1.8)
• 15–35 years	1,092	211 (19.3)		1.7 (1.3–2.3)	1.7 (1.2–2.4)
• 36 years and over	549	58 (10.6)	0.00	1	1
**Contact type**					
• Non-household contact	603	76 (12.6)		1	1
• Household contact	1,532	266 (17.4)	0.00	1.5 (1.1–2.0)	1.6 (1.1–2.2)
**Duration of close association**					
• 0–10 years	762	135 (17.7)		1	1
• 11–20 years	690	95 (13.8)		0.7 (0.5–1.0)	1.0 (0.7–1.3)
• 21 years or more	683	112 (16.4)	0.12	0.9 (0.7–1.2)	0.7 (0.5–1.0)
**Operational classification of Index cases**					
• Paucibacillary (PB)	565	76 (13.5)		1	1
• Multibacillary (MB)	1,57	266 (16.9)	0.05	1.3 (0.9–1.9)	1.4 (0.9–2.0)
**Sum of family BIs** *					
• 0–0.9	731	103 (14.1)		1	1
• 1.0–2.9	568	77 (13.6)		1.0 (0.7–1.4)	1.0 (0.7–1.6)
• 3.0–5.5	836	162 (19.4)	0.00	1.5 (1.1–2.0)	1.7 (1.2–2.3)

Notes:

1 Based on chi-squared tests.

2 Adjustment for clustering and age, gender, presence of BCG scar, type of association with IC, length of time of close association with IC, and sum of family BIs.

3 Confidence interval.

*: Bacteriological indexes.

**Figure 1:**
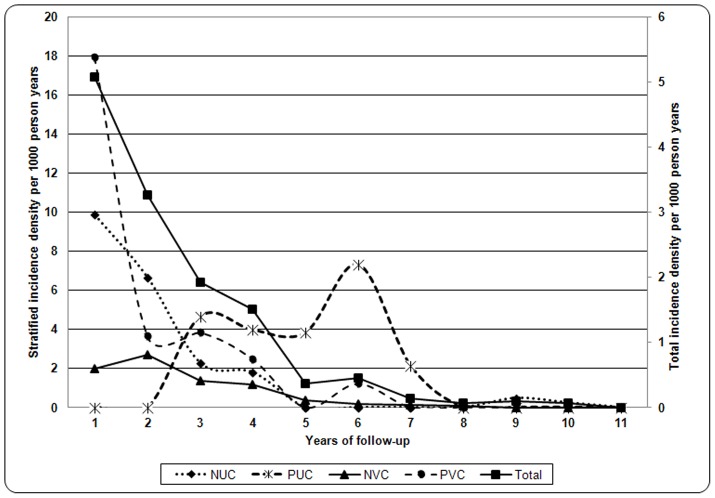
Global and stratified incidence density of leprosy cases according to PGL-I serology and BCG vaccination.

The contacts were followed for an average of 5.1±3.98 years (range: 0.21–18 years). During the follow-up period, 60 (28.1/1,000 PYs) new cases of leprosy were diagnosed at an incidence density of 5.08/1,000 PYs. Most of the cases (90%; 54/60) were contacts of MB patients. The average latency of detection of the new cases after the initial examination was 2.8 years (range: 3 months-10.5 years). Only 11 cases were detected during the first year after initial examination. The rate of detection declined steeply between the first and fourth years after the initial examination of contacts (Figure 1, solid line).

The incidence density of leprosy varied according to the BCG vaccination status and serology result (Figure 1, broken lines). PVCs and NUCs had the highest incidences of leprosy during the first year of follow-up at 17.9/1,000 PYs and 9.9/1,000 PYs, respectively. The effectiveness of the BCG vaccination was identified at the 2-year follow-up, rapidly reducing the incidence density to 2.5/1,000 PYs. However, the incidence density in NUCs did not begin to decrease until the third year. In addition, the incidence density was low during the initial years of follow-up in NVCs and progressively decreased, reaching 0 at 5 years of follow-up. Conversely, no cases of leprosy were diagnosed in the PUC group during the first 2 years of follow-up. However, the incidence density progressively increased in this group of contacts, with the highest values identified in the sixth year of follow-up. All of the groups converged to zero incidence during the 11th year of follow-up.

Leprosy diagnosis was strongly associated with PGL-I seropositivity. A significantly higher (χ^2^ = 11.2; p<0.01) proportion of incident cases was detected among PGL-I (+) contacts (5.6%, 19/342) during the follow-up period compared with PGL-I (−) contacts (2.3%; 41/1,793). PGL-I (+) contacts presented a 3.2-fold (95% CI: 1.6–6.1) higher risk for leprosy compared with PGL-I (−) contacts.

Stratification by vaccination status showed that the rate of developing leprosy was 1.8 times higher among unvaccinated than vaccinated contacts (8.3/4.6; p = 0.03). Among PGL-I (+) contacts, BCG vaccination after IC diagnosis increased the adjusted rate of developing clinical manifestations of leprosy (aRR = 4.1; 95% CI: 1.8–8.2) compared with the PGL-I (+) unvaccinated contacts (aRR = 3.2; 95% CI: 1.2–8.1).

Contacts aged 15–35 years showed a significantly (p<0.01) higher proportion of seropositivity (19.0%) compared with children (14.8%) and contacts aged >35 years (10.6%) (Table 1). Interestingly, after BCG vaccination, the 15- to 35-year-old age group presented a significantly (p = 0.02) lower rate of leprosy (2.5/1,000 PYs) compared with vaccinated PGL-I (+) children (6.6/1,000 PYs) and contacts over 35 years of age (6.8/1,000 PYs) (Table 2). In unvaccinated contacts, long periods of association with the IC and a high family BI were associated with the development of leprosy (Table 2).

**Table 2:** Adjusted rate ratios among leprosy contacts, stratified by BCG vaccination after index case diagnosis.

		BCG vaccine given subsequent to index case diagnosis				
	Vaccinated			Not vaccinated		
Covariates	Cases	Rate	Adjusted^2^ rate	Cases	Rate	Adjusted^2^ rate
	PYs^1^	Per	Ratios	PYs^1^	Per	Ratios
		1,000	(95%CI)^3^		1,000	(95%CI)^3^
		PYs			PYs	
**PGL-I status**	41/8,939	4.6	-	19/2,299	8,3	-
• PGL-I Positive	13/919	14.1	4.1 (1.9–8.8)	6/294	20.4	3.2 (1.2–8.1)
• PGL-I Negative	28/7,625	3.7	1.0	13/1,942	6.7	1.0
**Previous BCG**						
• BCG scar present	23/5,572	4.1	1.0 (0.4–2.3)	9/1,199	7.5	0.9 (0.3–2.1)
• No BCG scar	18/3,367	5.3	1.0	10/1,100	9.1	1.0
**Age group**						
• 0–14 years	11/1,679	6.6	2.7(1.1–6.9)	4/478	8.4	2.6 (0.5–12.3)
• 15–35 years	11/4,469	2.5	1.0	9/1,305	6.9	1.0
• 36 years and over	19/2,792	6.8	2.9 (1.2–7.0)	6/516	11.6	1.5 (0.7–3.7)
**Contact type**						
• Non-household contact	6/2,671	2.3	1.0	3/655	4.6	1.0
• Household contact	35/6,322	5.5	2.7 (1.1–6.4)	16/1,644	9.7	1.7 (0.4–7.9)
**Duration of close association**						
• 0–10 years	14/2,928	4.8	1.0	2/675	3.0	1.0
• 11–20 years	11/3,202	3.4	0.9 (0.4–2.0)	7/963	7.3	4.1 (0.8–20.2)
• 21 years or more	16/2,809	5.7	1.2 (0.5–2.9)	10/661	15.1	11.0 (1.7–71.2)
**Sum of family BIs** *						
• 0–2.5	6/5,753	1.0	1.0	6/1,309	4.6	1.0
• 2.6–3.5	10/1,172	8.5	9.3 (3.4–5.5)	4/533	7.5	1.1 (0.3–3.7)
• 3.6 and greater	25/2,013	12.4	10.6 (4.1–27.3)	9/457	19.7	4.1 (1.3–13.1)

1 Person years.

2 Adjustment for clustering and age, gender, presence of BCG scar, type of association with IC, length of time of close association with IC, and sum of family BIs.

3 Confidence interval.

*: Bacteriological indexes.

**Figure 2:**
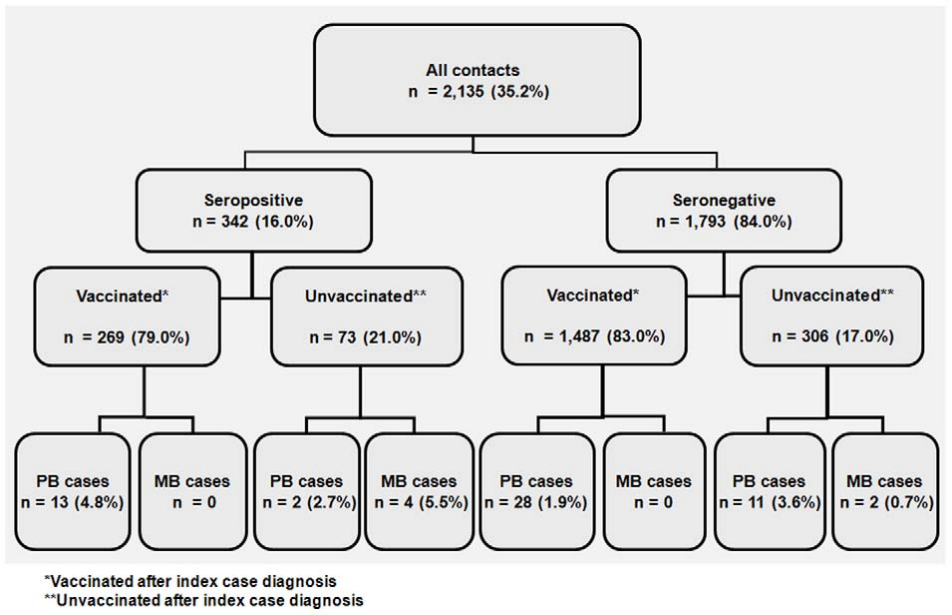
Leprosy cases by PGL-I result, BCG vaccination and clinical presentation in a cohort of contacts.

A significantly higher (p<0.01) proportion of PB leprosy cases was diagnosed in PVCs (4.8%; 13/269) compared with NVCs (1.9%; 28/1,487). All MB cases occurred among unvaccinated contacts and were diagnosed at a significantly higher rate (χ^2^ = 8.79; p = 0.03) in PUCs (5.5%; 4/73) than in NUCs (0.7%; 2/306) (Figure 2).

## Discussion

The predictive value of PGL-I seropositivity in the development of leprosy in contacts was analyzed as a method of identifying susceptible individuals among contacts of recently diagnosed patients. In addition, the possible interference of PGL-I seropositivity with the protective effect of BCG vaccination against leprosy was investigated.

The observed proportion of seropositivity (16.0%) was similar to that found in another study performed in Brazil [23]. The prevalence of seropositivity in this study showed associations with age and gender similar to those reported in other studies [6] [24]. According to Maddison *et al.* [25], females tend to demonstrate higher innate IgM levels than males, which may explain the high female seropositivity rate found in the present study. Independent of gender, seropositivity rates increased until young adulthood (15–35 years of age) and decreased in older adults, which is consistent with the general decrease in overall IgM levels observed with age [25]. It is well known that leprosy does not manifest preferentially in women or children, so these high levels are more likely explained by this common feature of the immune system rather than specifically reflecting differences in anti-PGL-I antibody levels in these groups.

The presence of a past BCG vaccination scar was associated with a higher seropositivity, but the association was weak and marginally significant when adjusted for covariates. This result corroborates findings of Baumgart *et al.* [26], who argued that BCG vaccination or exposure to tuberculosis or environmental mycobacteria could interfere with serological tests such as the PGL-I assay.

In the sample group of this study, PGL-I (+) contacts had a clear increased risk of developing leprosy. The independent effect of bacterial load, as measured by the familial BI, on the risk of developing leprosy among contacts is consistent with previous findings [19].

The increased incidence of leprosy observed in PVCs and NUCs during the first year of follow-up suggests subclinical infection. PVCs were partially benefited by BCG vaccination, as observed by Bagshawe *et al.* [18] in children, because they had insufficient time to build their immune capability to fight *M. leprae* but managed to avoid MB leprosy infection. BCG vaccination induces an increase in interferon-gamma (IFN-γ) production, which is highest among previously vaccinated individuals and those exposed to environmental mycobacteria [27]. Thus, the contacts' immune systems are predisposed to a cellular response that is effective against *M. leprae* [28]. IFN-γ production in response to *M. leprae* antigens is a measure of the ability to mount an effective immune response against the pathogen [29]. Thus, the lack of immune response among contacts exposed to the infectious agent could indicate susceptibility, as posited by Sampaio *et al.* [30]. The applicability of PGL-I testing for early diagnosis of clinical cases thus remains uncertain.

The overall decline in the incidence density of leprosy in contacts observed after the first year of diagnosis of the ICs, as observed by other authors [31], [32], could result from the treatment of index and co-prevalent cases. MDT seems to decrease in infectiousness over time. However, Groenen *et al.* [14] observed a mean yearly incidence rate of PB leprosy of 0.34%, with little variation during 4 years of follow-up. This difference may be explained by the hyperendemicity of the population studied by the latter authors and the irregular use of treatment by the patients.

The variation in the incidence density of leprosy according to BCG vaccination status and serological profile indicates that multiple factors are involved in the development of clinical overt leprosy in contacts. Together with early diagnosis and the treatment of ICs and co-prevalent cases, preventive measures such as contact evaluation, health education and immunization can prevent the transmission of leprosy.

Interestingly, the early peak in incidence and reduced infection levels in young adults, which reflect constitutional, age-related changes in the immune system [33], were only observed in the vaccinated group. Although the highest leprosy rates were expected among contacts aged 15–35 years, i.e., in the age group with the highest seropositivity, this group of contacts had the lowest incidence rate of leprosy after BCG vaccination. Regardless of their anti-PGL-I serological status, children are more susceptible than adults to acquiring leprosy infection and developing overt leprosy due to their incompletely developed immune systems and close and prolonged contact with possible intra-family sources of infection [34], [35]. Additionally, because BCG vaccination induces IFN-γ production [27], the strong immune response in young adults will control subclinical infection if present.

The known long incubation period of the disease was confirmed in the present study, as most of the MB cases occurred in PUCs (4/6) after the second year of follow-up. However, in the Yalisombo Study Group [14], the only MB case among the 13 incident cases in a 4-year cohort of 3207 contacts was diagnosed during the first year of follow-up. However, because the present study cohort was alerted to early signs of the disease, the contacts' awareness and subsequent detection of leprosy signs may have contributed to the high proportion of PB cases.

A major limitation of the present study was the use of a non-probabilistic sample group obtained at a reference leprosy center under routine conditions. In addition, the sample group may have had a selection bias toward older individuals, as children did not usually provide blood samples. However, the group in this study included contacts with a wide range of social and demographic characteristics who lived in a medium endemic region, which is similar to many settings in Brazil. Although it was not possible to ascertain the number of deaths during follow-up, the mortality rate due to leprosy is almost negligible within this age group [36]. In the ML flow test used to evaluate the presence/absence of antibodies against PGL-I, a precise distinction between positivity and negativity is sometimes difficult to ascertain. A misinterpretation of results due to grading from 0–4 could, in part, explain the finding of PB cases among the seronegative contacts.

It is well known that contacts of leprosy patients are at higher risk of developing leprosy and may even constitute a source of infection in the community at large [37]. In regions where no interventions are undertaken, contacts producing antibodies against *M. leprae* (corresponding to the PUCs in the present study) can be considered to be the main indicators of the maintenance of leprosy's endemic status. Nevertheless, early and effective interventions for contacts will affect the disease burden, leading to exhaustion of cases after 10 years of IC diagnosis.

The present study confirms that contact surveillance and health education combined with BCG vaccination remain important strategies for leprosy control. The fact that the highest rate of leprosy cases was found among PGL-I (+) unvaccinated contacts justifies targeting this specific group for close monitoring. Furthermore, it is highly recommended that PGL-I (+) contacts and contacts with high familial BIs be monitored regardless of serological response. Targeting these groups for a more focused and specific approach such as chemoprophylaxis could make this intervention strategy more cost-effective.

## Supporting Information

Checklist S1 **STROBE checklist.**
(DOC)
